# Strengthening climate-health literacy through sustainability education among dental students: a quasi-experimental evaluation

**DOI:** 10.1186/s12909-026-08987-1

**Published:** 2026-03-11

**Authors:** Ömer Faruk Sönmez, Grisela Prenga, Aysel Başer, Sema Belli, Gül Çelik

**Affiliations:** 1 School of Medicine and Population Health, University of Sheffield, Sheffield, UK; 2 Vocational School Health Services, Kırıkkale University, Kırıkkale, Türkiye; 3 École des hautes études en santé publique, Rennes, France; 4 Department of Medical Education, Faculty of Medicine, Izmir Democracy University, İzmir, Türkiye; 5 Department of Endodontics, Faculty of Dentistry, Selcuk University, Konya, Türkiye; 6 Department of Endodontics, Faculty of Dentistry, Izmir Democracy University, İzmir, Türkiye

**Keywords:** Climate-health literacy, Sustainability, Dental education, Quasi-experimental, CCPS-HARPS, Oral health

## Abstract

**Introduction:**

Health systems both face escalating climate risks and contribute to emissions. Dentistry can mitigate impacts via prevention-oriented, low-carbon, and resilient practice, yet climate-health literacy is inconsistently taught. We evaluated whether a curriculum-embedded sustainability module improves climate-health literacy and action readiness among dental students.

**Methods:**

We conducted a quasi-experimental, two-site, parallel-group evaluation with pre- and post-testing in Türkiye (spring 2023–2024). Fourth-year students at Selcuk University (intervention; 14-week sustainability module) and Izmir Democracy University (control; standard curriculum) completed surveys at baseline (week 0) and post-teaching (weeks 14–15). Primary outcomes were the Climate Change Perceptions Scale for Health and Related Professionals and Students (CCPS-HARPS) total and subscales: Awareness & Consciousness, Health & Climate Change, and Advocacy & Experience. Study-specific Oral Health and Willingness-to-Act scales captured discipline-specific perceptions and readiness. Analyses included within-group paired tests, ANCOVA of post-test scores adjusted for baseline (and GPA), assumption checks with interaction probing via estimated marginal means, reliability (Cronbach’s α), and multivariable linear regression with demographic covariates.

**Results:**

Baseline included 67 control and 83 intervention students (post-test n = 65 and n = 75, respectively). Internal consistency was acceptable-to-excellent (α = 0.64–0.89 pre; 0.79–0.92 post). In the control group, Awareness (Δ=−0.17, p=0.017), Advocacy (Δ=−0.44, p<0.001), and CCPS Total (Δ=−0.16, p=0.004) declined. The intervention group improved in Health & Climate (Δ=+0.20, p=0.002), CCPS Total (Δ=+0.12, p=0.025), and Willingness-to-Act (Δ=+0.18, p=0.009), with a borderline increase in Oral Health (Δ=+0.12, p=0.061). Adjusted post-tests favored the intervention for Awareness (p=0.025; η²ₚ=0.036), Health & Climate (p=0.001; 0.075), Advocacy (p=0.002; 0.066), CCPS Total (p<0.001; 0.091), and Willingness-to-Act (p=0.006; 0.053); Oral Health was not significant (p=0.190; 0.013). Slope non-homogeneity for Health & Climate and CCPS Total showed larger benefits at lower baselines (e.g., CCPS Total difference 0.37 at 25th percentile, p<0.001), indicating gap-narrowing effects.

**Conclusions:**

A semester-long, curriculum-embedded sustainability module produced small-to-moderate gains in climate-health literacy and action readiness among dental students, with the greatest benefits for those starting lower. Findings support integrating assessed, discipline-specific sustainability learning across dental curricula to enable climate-smart, prevention-oriented oral healthcare.

**Supplementary Information:**

The online version contains supplementary material available at 10.1186/s12909-026-08987-1.

## Introduction

Human activity has raised atmospheric greenhouse gases since the industrial era, warming the planet and disrupting weather, water, and ecosystems at a pace that threatens population health worldwide. The World Health Organization (WHO) calls climate change the greatest health risk of this century, because it undermines clean air and water, food security, and stable living conditions that keep communities healthy. Scientific assessments detail how rising temperatures, shifting precipitation, sea level rise, and more frequent extremes intensify heat illness, respiratory and cardiovascular disease, injuries, mental health harms [1], and infectious disease transmission [2, 3].

These health impacts are not abstract. They are already measurable across continents, with heightened risks for children, older adults, and communities with fewer resources. Annual global tracking links climate exposure to heat related mortality, degraded air quality, food and water stress, and shifting vector ranges, with health systems facing rising demand as hazards intensify [4]. The evidence base also shows why early and sustained action matters. Delays raise cumulative emissions and lock in higher health burdens, while near term mitigation and adaptation deliver rapid health co-benefits through cleaner air, safer housing, and more resilient services [5].

Health systems are material and energy intensive and contribute meaningfully to global warming. Best estimates attribute roughly 4 to 5% of global greenhouse gas emissions to the health sector, with the majority arising from supply chains rather than on-site operations. Procurement accounts for about two thirds to three quarters of the footprint, followed by energy use and smaller contributions from direct emissions, including anesthetic gases [6].

The WHO operational framework specifies governance, risk assessment, facilities, energy, water, waste, supply chains, transport, and information systems as levers for action, emphasizing routine measurement and integration with quality and safety agendas. Core strategies follow from this evidence base; reduce demand through efficiency and prevention, electrify and procure renewable power, substitute high-impact clinical products and gases where clinically appropriate, redesign care and logistics to limit travel, and reform purchasing toward durable, repairable, and reusable goods with verified life-cycle data. Embedding these steps in curricula and professional development enables clinical leaders to normalize climate stewardship as part of safe, high-value care [7, 8].

Health system performance, once framed mainly around equitable access, workforce, service delivery and finance, now also recognises environmental sustainability and climate resilience as integral to quality and safety [9]. Because the sector both bears a substantial greenhouse gas footprint and faces escalating climate risks, planning and operating low-carbon, climate-resilient services has become a systems imperative rather than an optional add-on [10, 11]. This shift is reshaping professional competence: health workers require climate and health literacy that weaves together basic climate science and ecological change with the pathways from environmental disruption to health outcomes, a clear understanding of the sector’s own contributions and mitigation levers, and the practical skills needed for sustainable clinical practice, coupled with the communication capacities to serve as trusted sources for patients and communities [12]. Reflecting this evolution, emerging curricula and professional frameworks across health disciplines are moving toward vertically integrated, assessed learning outcomes across undergraduate education, postgraduate training and continuing professional development [13, 14].

Climate change affects oral health through clear environmental and social pathways. Heat and dehydration reduce salivary flow and increase caries risk; longer allergy seasons and poor air quality worsen mouth-breathing, xerostomia, and periodontal inflammation. Disruptions to food and water security after floods, storms, or droughts hinder hygiene and timely care, amplifying untreated infections. These burdens fall most on children, older adults, and resource-limited communities, widening existing oral health inequities [15].

Dental care is also an emissions source and should be organized accordingly. Prevention and early detection are the strongest mitigation levers because they avert procedures and repeat visits. When treatment is needed, practices can cut travel and material intensity by combining appointments, using tele-dentistry where appropriate, and adopting same-day digital workflows. Facility actions include efficient electrified heating, ventilation, and air conditioning, renewable electricity, water-saving vacuum systems, and robust waste protocols (digital radiography, safe amalgam capture, selective reusables). Climate-resilient operations help maintain access during extreme weather, aligning quality care with environmental responsibility [16, 17] .

Carbon footprint assessments indicate that dentistry is a non-trivial contributor to health-sector emissions. In England, carbon modelling of NHS dental services estimated total emissions of ~ 675,706 tCO₂e/year (2013/14), with the largest shares arising from patient and staff travel (~ 64.5% combined), followed by procurement (~ 19%) and energy use (~ 15%); smaller contributions came from nitrous oxide, waste, and water [18]. From the same Public Health England Report, at the procedure level, common care episodes range from approximately ~ 5.5 kgCO₂e for an examination-based course of treatment (including radiograph/fluoride varnish), to ~ 23.3 kgCO₂e for endodontic treatment, and ~ 58–71 kgCO₂e for dentures, illustrating how avoidable disease progression can substantially increase lifetime environmental impacts [18]. Beyond greenhouse gases, dentistry generates environmental burdens through clinical and packaging waste (notably single-use plastics and PPE) and dental materials, while mercury releases from dental amalgam waste remain a specific pollutant concern addressed through global commitments to phase down and ultimately end amalgam use.

Internationally, dentistry is beginning to translate sustainability principles into concrete professional guidance and practice initiatives. The FDI World Dental Federation has issued a global policy statement on Sustainability in Dentistry, encouraging prevention-focused, high-quality care that reduces environmental impact and embeds sustainability into professional responsibilities [19]. In parallel, implementation of the Minamata Convention on Mercury has driven practical waste-management measures in dentistry (e.g., encapsulated amalgam use and amalgam separators), supported by profession-led implementation guidance [20]. At the service and practice level, applied resources such as the Sustainable Dentistry Guide provide actionable recommendations for dental teams on travel, procurement, energy/water use, and waste reduction [21]. Additionally, practice-facing programmes such as the Eco-Dentistry Association’s GreenDOC certification have emerged to help dental clinics document and scale environmentally responsible workflows [22].

Education has emerged as a central component on tackling climate literacy among professionals. Survey work across the United Kingdom and Ireland indicates that most dental schools do not yet teach environmental sustainability, with 56% reporting no current provision and commonly cited barriers including time constraints and limited learning resources [23]. At the same time, regulators and professional bodies are creating momentum by embedding relevant learning outcomes and signalling future compliance expectations [24].

Complementary evidence from Greece shows that dental students display higher environmental awareness than practicing dentists and that moral commitment, recognition of climate change as a major problem, and attention to packaging and purchasing are associated with pro-environmental actions. These findings support targeted integration of sustainability into undergraduate curricula and continuing professional development [25].

Beyond curricula, professional roles are expanding toward advocacy, prevention, and service redesign. Recommendations emphasize educating patients and communities, collaborating with professional associations and policymakers, and aligning with value-based care models that prioritize prevention and coordinated records, thereby reducing avoidable treatment and associated emissions [26]. Commentaries warn that intensifying weather hazards are reshaping insurance markets and may raise premiums or limit coverage for practices in high-risk regions, with implications for access and equity [16]. More broadly, health and public health workforces benefit from clearly defined, competency-based professionalization pathways that align law, education, and continuing development to strengthen system performance [27].

Overall, the literature on oral health and dentistry in the context of climate change and sustainability converges on four priorities:

1. Reorient care toward prevention to lower disease burden, reduce visits, and conserve resources [28].
2. Decarbonise care pathways by reducing travel, adopting sustainable procurement, improving energy and water efficiency, minimising waste, curbing single-use plastics, and avoiding unnecessary mercury use [29].
3. Embed environmental sustainability and One Health competencies across undergraduate and continuing professional education, with practical, assessed learning outcomes [30, 31].
4. Plan for climate resilience in practice operations, including facilities, data systems, supply chains, and insurance [32].

Across the evidence base, quantitative models consistently identify prevention as the most impactful single lever for reducing dentistry’s environmental footprint. Accordingly, this article focuses on education, introducing and evaluating a curriculum intervention on environmental sustainability in dental training.

To situate environmental sustainability agenda in dental education, it is useful to ask how climate and sustainability education has been designed and evaluated across health professions, and what outcomes have been achieved. Recent programmes span electives, online modules, serious games, workshops within quality improvement teaching, and longitudinal curricular embedding across medicine, nursing, and dentistry [33]. For instance, Gomez et al. showed that a dedicated climate and health elective for preclinical medical students significantly increased confidence to explain climate health links, to apply sustainability principles in clinical practice, and to advocate for curricular inclusion, with post course gains across all knowledge and application items [34]. Leung et al. showed that an asynchronous module for dental hygiene students produced statistically significant improvements in knowledge and attitudes toward environmentally sustainable dentistry, with most learners reporting that the follow up assignment helped them apply concepts in clinic [35]. Dixon et al. showed that embedding environmental sustainability across an entire undergraduate dental curriculum yielded significant positive changes in awareness, knowledge, and general pro environmental attitudes at post intervention [36].

Similar studies have highlighted the importance of climate literacy among professionals. Clery et al. showed that integrating sustainability within quality improvement teaching created strong intentional value, enhancing engagement and prompting calls for sustainability to be integrated across the curriculum rather than siloed [37]. Stevens et al. showed that a novel serious game increased medical students’ knowledge and pro climate attitudes, while highlighting the need to address accompanying climate worry by building agency [38]. Elhoshy et al. showed that an online planetary health and sustainable healthcare course for medical students produced a significant pre to post improvement in test scores and very high satisfaction with course quality and instructor communication [39]. Çolak et al. showed that a climate change and health course for nursing students increased global warming knowledge and attitudes and modestly raised eco anxiety, with no measurable change in general environmental literacy [40]. Mohammed et al. showed that an awareness program in a nursing cohort significantly improved overall understanding, perceptions, and day to day pro environmental routines, with higher knowledge associated with more sustainable behaviors [41].

The participating dental programmes aim to graduate a general dentist aligned with Türkiye’s national competency and qualifications frameworks (DUÇEP; TYYÇ). Graduate outcomes emphasise competent, evidence-based diagnosis and treatment across core domains of dentistry, prevention and health promotion, safe management of clinical/medical emergencies, ethical and legal professionalism, effective communication with patients and the health system, and lifelong learning and quality improvement. Within this profile, sustainability is relevant not as an “add-on” topic but as an enabling competence that supports high-value preventive care and responsible clinical decision-making in material-, energy-, and waste-intensive dental services [42].

This study aims to evaluate whether a 14-week sustainability focused curriculum embedded module improves dental students’ climate and health related knowledge and awareness, perceived impacts, advocacy and experience, oral health considerations, and willingness to act, relative to a control group, by analyzing pre and post CCPS domain scores with adjustments for baseline differences and demographic covariates. Unlike much of the existing literature that centers on short courses, brief interventions, or educational games, this study evaluates a module embedded within the dental curriculum, providing evidence for a scalable and sustained approach.

## Methods

### Study design and setting

This was a quasi-experimental, two site, parallel group evaluation with pre-test and post-test measurement. Fourth year dental students at two public universities in Türkiye formed naturally occurring groups by institution. Selcuk University (SU) delivered a 14-week sustainability focused dentistry module during the spring term of the 2023 to 2024 academic year and served as the intervention site. Izmir Democracy University (IDU) continued the standard curriculum without structured sustainability content during the same term and served as the control site. No randomization or crossover occurred. Data were collected online at baseline in week 0 and again at the end of teaching in weeks 14 to 15 using the same instruments.

Both participating dental schools deliver an outcomes-based undergraduate dental programme aligned with Türkiye’s National Core Dental Education Program (DUÇEP) and the Türkiye National Competences Framework (TYYÇ)/Bologna learning-outcomes framework. Teaching is predominantly clinically oriented, with progressive preclinical preparation followed by increasing clinical responsibilities in later years. The intervention was implemented within this existing educational model and did not involve broader curriculum restructuring at either institution.

### Participant recruitment and eligibility

All enrolled fourth year students at both schools were invited to take part. Recruitment used brief in class announcements and departmental emails that provided an information sheet and a survey link. Inclusion criteria were age 18 years or older, current enrolment in the fourth year of the dental program, and provision of informed consent. There were no exclusions based on academic standing or prior exposure to climate topics. Participation was voluntary and unrelated to grades. Students who declined consent or did not complete the baseline survey were not enrolled. To link pre and post responses while preserving anonymity, the survey platform issued a unique code to each consenting student. The control and intervention cohorts had no shared teaching sessions during the term to minimize contamination.

### Sample size and power

The design targeted a census of both cohorts rather than a fixed sample size. The achieved baseline sample was 83 students in the intervention group and 67 students in the control group. 2 students from IDU and 8 students from SU have not participated int the post-test and they were not therefore included in study sample. A priori, this total sample of 150 provides at least 80% power at alpha 0.05 to detect a small to moderate between group effect in ANCOVA with one covariate, corresponding to Cohen f about 0.20 and partial eta squared about 0.04, assuming a moderate correlation between pretest and post-test scores. Within groups, samples of about 65 to 75 students provide at least 80% power to detect a paired pre to post effect of Cohen d about 0.30 under two tailed testing. The study was not powered to detect very small effects.

### Intervention

The intervention was a 14-week, curriculum embedded sustainability module delivered to fourth year students at Selcuk University during the spring term. The module aimed to build climate and health literacy and translate it to prevention oriented, low carbon, and resilient dental practice. Teaching combined brief lectures, case based discussion, and small group tasks within routine timetabled sessions. The full module descriptor, including weekly topics, learning objectives, and required activities, is provided in Supplementary File 1. Delivery fidelity was monitored through simple instructor checklists and attendance logs. For example, students completed a small-group ‘environmental hot spots’ exercise to identify major contributors to dental care’s footprint and propose feasible 4R (Reduce–Reuse–Recycle–Rethink) quality-improvement actions in clinic settings, and they participated in case-based discussions comparing biomimetic versus conventional restorative options while explicitly weighing prognosis/longevity alongside resource use and waste. The complete module descriptor and weekly activities are provided in Supplementary File 1.

### Outcomes assessed

The primary outcome set was the Climate Change Perceptions Scale for Health and Related Professionals and Students (CCPS HARPS), a validated instrument with three subscales which was developed and validated by Sonmez et al. in a different study [43],

1. Awareness and Consciousness
2. Health and Climate Change
3. Advocacy and Experience

Items are rated on a five-point Likert scale, reverse coded where appropriate, and averaged so higher scores indicate greater awareness or agreement. We examined the CCPS total mean score and each subscale at baseline and post intervention.

Two brief, study specific surveys were developed for this study and added to capture dental practice relevance and action readiness which can be accessed at Supplementary File 2:

- Oral Health perceptions scale focused on climate related risks and responses in dentistry
- Willingness to Act scale focused on readiness to change practice, educate patients, and engage in improvement or advocacy

Both used the same five-point Likert format with scoring aligned so higher values reflect greater perceived relevance or readiness. Internal consistency for all scales was evaluated at both time points. Primary analyses considered CCPS total, the three CCPS subscales, Oral Health, and Willingness to Act. Measurements were collected online at week 0 and weeks 14 to 15.

### Statistical analysis

All analyses were conducted in R (version 2024.12.1) using standard statistical and psychometric packages. Prior to analysis, item-level responses were checked for missing values and out-of-range entries. All items were coerced to numeric form, and reverse-coded items were recoded so that higher scores consistently indicated greater awareness or agreement. For each domain and for the total Climate Change Perception Scale (CCPS), mean composite scores were computed separately for the pre-test and post-test. The internal consistency of each scale was evaluated using Cronbach’s α at both measurement points to verify the reliability of the domain scores as seen in most scale adaptation and development studies [44].

Baseline analyses were performed to assess group comparability before the intervention. Demographic variables (age, gender, marital status, GPA category, and previous education or training related to sustainability) and baseline domain scores were summarized by mean (SD) or frequency (%). Independent-samples t-tests (with Welch’s correction when variances were unequal) were used for continuous variables, and Chi-square or Fisher’s exact tests were used for categorical variables. Cohen’s d and Cramér’s V were calculated as effect sizes to describe the magnitude of baseline differences. Variables that differed significantly between groups at baseline were later entered as covariates in the adjusted analyses.

Within each group, pre–post differences were analyzed to assess change over time. Paired-samples t-tests were used when assumptions of normality were met; otherwise, the Wilcoxon signed-rank test was used as a non-parametric alternative. For each domain, the direction and magnitude of change were summarized by mean difference, *p*-value, and effect size (Cohen’s d for paired samples, corrected for dependence). These analyses described whether participants improved or declined independently of the other group.

Between-group intervention effects were examined using Analysis of Covariance (ANCOVA) for each domain and the overall CCPS score. In each model, the post-test score was entered as the dependent variable, group (control vs. intervention) as the fixed factor, and the corresponding pre-test score as a covariate to adjust for baseline differences.

Model assumptions were checked:

- Linearity of the relationship between the covariate and outcome was confirmed by inspecting model residuals;
- Homogeneity of variance across groups was tested using Levene’s test; and
- Homogeneity of regression slopes was evaluated by adding a group × pre-test interaction term to the model.

If the interaction term was non-significant (*p* > 0.05), the standard ANCOVA model was interpreted. If the assumption was violated (*p* < 0.05), the interaction model was retained and simple-effects probing was performed using estimated marginal means (EMMs) at the 25th, 50th, and 75th percentiles of the pre-test distribution to explore how the group difference varied by baseline level. Adjusted post-test means with 95% confidence intervals were obtained from the EMMs. Effect sizes for ANCOVA were reported as partial eta-squared (η²ₚ), interpreted as small (≈ 0.01), medium (≈ 0.06), or large (≥ 0.14).

Finally, exploratory multiple linear regression models were fitted for each domain to confirm the robustness of results when including potential demographic predictors (age, gender, GPA, marital status, and prior education) alongside group and pre-test score.

All analyses were two-tailed with an alpha level of 0.05. Results are presented with their corresponding test statistics, *p*-values, and effect sizes.

## Results

Baseline characteristics were comparable across groups except for GPA distribution (Table 1). The control cohort included 67 students and the intervention cohort included 83 students. Mean age did not differ between groups (22.1 years, SD 1.7 versus 22.2 years, SD 1.8; *p* = 0.13). Gender, marital status, and prior education or training were also similar (all *p* > 0.05). GPA categories differed significantly, with a higher proportion of intervention students in the upper GPA bands (*p* = 0.001). GPA was therefore included as a covariate in adjusted analyses.

**Table 1:** Baseline characteristics of participants by group

Characteristic	Control (*N* = 67)	Intervention (*N* = 83)	*p*-value
Age (years)			0.13
	22.1 (1.7)	22.2 (1.8)	
Gender			0.34
Men	22 (33%)	32 (39%)	
Women	45 (67%)	51 (61%)	
Marital status			0.42
Single	61 (91%)	72 (87%)	
Married	6 (9%)	11 (13%)	
GPA category			**0.001**
2.00–2.50	15 (22%)	7 (8%)	
2.51–3.00	28 (42%)	26 (31%)	
3.01–3.50	17 (25%)	34 (41%)	
≥ 3.51	7 (10%)	16 (19%)	
Prior education/training			0.57
Yes	10 (15%)	15 (18%)	
No	57 (85%)	68 (82%)	

Values are mean (SD) or *n* (%). *p* values from independent samples t test for age and from Pearson chi square or Fisher exact test for categorical variables, as appropriate

Within group change analyses showed contrasting patterns between cohorts. In the control group, mean scores declined from pretest to posttest in Awareness and Consciousness (Delta − 0.17; t(64) = 2.45, *p* = 0.017; d_av = − 0.30), Advocacy and Experience (Delta − 0.44; t(64) = 3.75, *p* < 0.001; d_av = − 0.47), and the CCPS Total score (Delta − 0.16; t(64) = 2.97, *p* = 0.004; d_av = − 0.37), with no significant change in Health and Climate, Oral Health, or Willingness to Act (all *p* > 0.05). In contrast, the intervention group improved in Health and Climate (Delta + 0.20; t(74) = − 3.22, *p* = 0.002; d_av = + 0.37), CCPS Total (Delta + 0.12; t(74) = − 2.29, *p* = 0.025; d_av = + 0.26), and Willingness to Act (Delta + 0.18; t(74) = − 2.68, *p* = 0.009; d_av = + 0.31), with a marginal increase in Oral Health (Delta + 0.12; *p* = 0.061). Changes in Awareness and Consciousness and in Advocacy and Experience were not significant in the intervention group (both *p* = 0.31). Full descriptive statistics and tests are shown in Table 2.

**Table 2:** Within group pretest to posttest change by domain

Domain	Pre-test M (SD)	Post-test M (SD)	Δ (Post–Pre)	t(df)	*p*	Cohen’s dav
Control						
Awareness (F1)	4.25 (0.53)	4.08 (0.49)	–0.17	2.45	**0.017**	–0.30
Health & Climate (F2)	3.79 (0.61)	3.73 (0.58)	–0.06	0.98	0.33	–0.12
Advocacy (F3)	4.04 (0.54)	3.60 (0.63)	–0.44	3.75	< **0.001**	–0.47
CCPS Total	3.99 (0.45)	3.83 (0.44)	–0.16	2.97	**0.004**	–0.37
Oral Health	4.12 (0.54)	4.08 (0.50)	–0.04	0.63	0.53	–0.08
Willingness	3.49 (0.61)	3.45 (0.60)	–0.04	0.37	0.71	–0.05
Intervention						
Awareness (F1)	4.20 (0.50)	4.26 (0.45)	+ 0.06	–1.02	0.31	+ 0.12
Health & Climate (F2)	3.82 (0.60)	4.02 (0.53)	+ 0.20	–3.22	**0.002**	+ 0.37
Advocacy (F3)	3.88 (0.55)	3.96 (0.52)	+ 0.08	–1.03	0.31	+ 0.12
CCPS Total	3.97 (0.44)	4.09 (0.41)	+ 0.12	–2.29	**0.025**	+ 0.26
Oral Health	4.04 (0.48)	4.16 (0.42)	+ 0.12	–1.90	0.061	+ 0.22
Willingness	3.58 (0.59)	3.76 (0.51)	+ 0.18	–2.68	**0.009**	+ 0.31

Between group comparisons at posttest, adjusted for baseline scores, showed consistent advantages for the intervention across most outcomes (Table 3). Significant group effects were observed for Awareness and Consciousness (*p* = 0.025; partial eta squared = 0.036), Health and Climate (*p* = 0.001; 0.075), Advocacy and Experience (*p* = 0.002; 0.066), CCPS Total (*p* < 0.001; 0.091), and Willingness to Act (*p* = 0.006; 0.053). Oral Health did not differ significantly between groups (*p* = 0.190; 0.013). Adjusted means were higher in the intervention arm for every domain; for example, CCPS Total was 4.10 in the intervention group versus 3.81 in the control group, indicating small to moderate educational effects.

**Table 3:** Adjusted posttest comparisons by group (ANCOVA)

Domain	*p* (Group Effect)	Partial η²	Adjusted Mean (Control)	Adjusted Mean (Intervention)
Awareness (F1)	**0.025**	0.036	4.06	4.27
Health & Climate (F2)	**0.001**	0.075	3.73	4.02
Advocacy (F3)	**0.002**	0.066	3.55	4.00
CCPS Total	<** 0.001**	0.091	3.81	4.10
Oral Health	0.190	0.013	4.05	4.18
Willingness	**0.006**	0.053	3.48	3.73

To test robustness beyond ANCOVA, we fit multiple linear regression models for each outcome with posttest score as the dependent variable and group, the corresponding pretest score, age, gender, GPA category, marital status, and prior education or training as predictors. Group remained a significant positive predictor for Awareness and Consciousness, Health and Climate, Advocacy and Experience, CCPS Total, and Willingness to Act, but not for Oral Health (Table 4). Model R² values ranged from 0.21 to 0.37, indicating that the predictors explained 21 to 37% of variance in posttest outcomes. These results are directionally consistent with the ANCOVA findings.

**Table 4:** Multivariable regression predicting posttest scores by domain

Domain	*R*²	β (Group: Intervention)	SE	t	*p*
Awareness (F1)	0.22	0.21	0.10	2.15	**0.033**
Health & Climate (F2)	0.35	0.28	0.09	3.03	**0.003**
Advocacy (F3)	0.21	0.42	0.16	2.73	**0.007**
CCPS Total	0.32	0.28	0.08	3.33	**0.001**
Oral Health	0.23	0.16	0.10	1.54	0.127
Willingness	0.37	0.25	0.09	2.62	**0.010**

β is the unstandardized coefficient for group coded 1 for intervention and 0 for control. Positive β indicates higher adjusted posttest scores in the intervention group after controlling for covariates. R² is the coefficient of determination for each model

Assumption checks indicated a violation of homogeneity of regression slopes for Health and Climate (F2) and CCPS Total. We therefore retained the group by pretest interaction and probed simple effects using estimated marginal means at the 25th, 50th, and 75th percentiles of the baseline distribution. As shown in Table 5, intervention effects were largest among students with lower baseline scores and diminished with higher baseline levels. For F2, the adjusted posttest difference favored the intervention by 0.42 at the 25th percentile (SE 0.11, 95% CI 0.19 to 0.65, *p* < 0.001), 0.28 at the 50th percentile (SE 0.10, 95% CI 0.09 to 0.47, *p* = 0.004), and 0.11 at the 75th percentile (SE 0.09, 95% CI − 0.06 to 0.28, *p* = 0.18). A similar pattern held for CCPS Total, with differences of 0.37 (SE 0.09, 95% CI 0.19 to 0.55, *p* < 0.001), 0.25 (SE 0.08, 95% CI 0.09 to 0.41, *p* = 0.002), and 0.09 (SE 0.08, 95% CI − 0.07 to 0.25, *p* = 0.26) at the 25th, 50th, and 75th percentiles, respectively. These findings indicate that the curriculum produced the greatest gains for students who began with lower initial awareness or understanding.

**Table 5:** Simple effects of group at selected baseline percentiles from interaction models

Domain	Pre-test Level	Group Difference (Estimate)	SE	95% CI	*p*
Health & Climate (F2)	Low (25th)	**0.42**	0.11	(0.19, 0.65)	< **0.001**
	Medium (50th)	0.28	0.10	(0.09, 0.47)	**0.004**
	High (75th)	0.11	0.09	(–0.06, 0.28)	0.18
CCPS Total	Low (25th)	**0.37**	0.09	(0.19, 0.55)	< **0.001**
	Medium (50th)	0.25	0.08	(0.09, 0.41)	**0.002**
	High (75th)	0.09	0.08	(–0.07, 0.25)	0.26

Internal consistency for all scales was acceptable to very good. As can be seen Table 6, Cronbach’s α coefficients ranged from 0.64 to 0.89 at pre-test and from 0.79 to 0.92 at post-test, indicating that all domains demonstrated reliable internal coherence. Reliability improved for most scales after the intervention, most notably for Advocacy and Experience (α = 0.64 to 0.79), suggesting that students’ responses became more consistent and conceptually aligned following the course.

**Table 6:** Internal consistency of study scales at pretest and posttest

Domain	Cronbach’s α (Pre)	Cronbach’s α (Post)
Awareness & Consciousness (F1)	0.83	0.88
Health & Climate Change (F2)	0.81	0.88
Advocacy & Experience (F3)	0.64	0.79
CCPS Total	0.89	0.92
Oral Health	0.84	0.87
Willingness to Act	0.73	0.80

## Discussion

A quasi-experimental design was appropriate because intact institutional cohorts could not be randomized without disrupting timetables and assessment structures. In such settings, pretest–posttest comparisons with statistical adjustment provide defensible estimates of educational impact, provided key assumptions are checked and reported [45, 46]. The CCPS-HARPS was selected as the primary outcome because it is a health-profession-specific, validated measure with a three-factor structure and excellent internal consistency and fit indices, designed to capture awareness, perceived effects, and health links of climate change among students and professionals [43, 47].

Two brief add-on surveys addressed domains not fully covered by CCPS but central to competency-based education in sustainability. First, willingness to act was included because intention is a proximal predictor of behaviour in well-supported behavioural theory and meta-analytic evidence, making it a meaningful bridge between knowledge gains and practice change [48]. Second, an oral-health–specific scale captured climate–dentistry interfaces that general climate-health tools do not, consistent with calls to tailor sustainability education to discipline-specific workflows and environmental footprints in dentistry [13] .

This quasi-experimental evaluation across two Turkish dental schools shows that a curriculum embedded sustainability module can produce meaningful gains in climate and health literacy among fourth year students. After one semester of teaching integrated into routine timetables, adjusted post-test means favoured the intervention for Awareness and Consciousness, Health and Climate, Advocacy and Experience, the CCPS Total, and Willingness to Act. Effect sizes were small to moderate which is noteworthy for a single term intervention delivered in parallel with normal clinical commitments. Oral Health specific perceptions increased in the intervention cohort but the between group difference was not significant, which suggests either high initial familiarity with climate links to oral diseases and services or a need for deeper practice anchored content to move this domain further.

The pattern of within group change supports a genuine educational signal rather than regression to the mean. Control students showed declines from pre-test to post-test in Awareness and Consciousness, in Advocacy and Experience, and in the CCPS Total, with no improvements in the other domains. In contrast, intervention students improved in Health and Climate, in the CCPS Total, and in Willingness to Act, with a near significant rise in Oral Health. Awareness and Advocacy did not change within the intervention group, which can be due to high starting values. The results strengthen the interpretation that structured teaching helped sustain and advance climate literacy during a busy clinical term.

Between group comparisons that adjusted post-test scores for baseline values showed coherent advantages for the intervention cohort across five of the six outcomes. The size of these adjusted differences is consistent with the broader health professions education literature, where short courses, online modules, and serious games typically yield small to moderate pre to post gains in knowledge, perceived impacts, and willingness to engage in sustainability focused practice and advocacy [34–41]. Our study adds that gains can be achieved when sustainability content is embedded as core curriculum rather than offered as an optional add on, and that benefits extend beyond knowledge into advocacy orientation and action readiness.

Multivariable regression models that included age, gender, GPA category, marital status, and prior exposure to sustainability content confirmed the robustness of these findings. Group membership remained a significant positive predictor for the same five outcomes while Oral Health was again non-significant. Model R squared values from 0.21 to 0.37 indicate that baseline score and a small set of covariates explain a meaningful proportion of post-test variance, while still leaving room for unmeasured contributors.

Interaction models illuminate for whom the module worked best. For Health and Climate and for the CCPS Total, the assumption of homogeneous regression slopes was not met, and simple effects showed the largest intervention advantages among students at lower baseline percentiles, with diminished or null differences at higher starting points. This gradient implies that embedding teaching across a semester helps close initial literacy gaps within a cohort. That pattern is educationally important because programs seek to bring all graduates to a defined threshold of competence, not only to raise already engaged students.

Although this study did not measure affective outcomes such as climate worry or eco-anxiety, the overall pattern aligns with work positioning prevention and systems literacy as the strongest levers for decarbonizing oral health care while maintaining access and quality [49]. A semester-long, discipline-specific module likely operated through three reinforcing mechanisms: repeated exposure over fourteen weeks that supported retrieval and transfer across cases; grounding in dental workflows that turned general climate–health concepts into concrete clinical choices such as appointment clustering, appropriate use of teledentistry, digital radiography, selective reusables, and water-saving vacuum systems, consistent with the FDI and dental authorities’ recommendations [50] ; and structured, feasible tasks that likely increased students’ sense of agency, a theorized bridge from knowledge to action in prior evaluations.

Oral Health did not show a significant adjusted between-group difference despite a small, borderline within-group rise in the intervention cohort. This is plausible given the scale’s focus on climate impacts on oral conditions rather than on sustainable practice behaviors emphasized in the module, the high baseline means that constrained headroom, and the presence of conceptually nuanced items that may elicit more cautious agreement after teaching. Future iterations should pair this perception scale with brief, practice-anchored content on climate–oral pathways and with objective indicators of enactment, so that changes in understanding and behaviour can be captured together.

The small declines in the control group’s Awareness, Advocacy, and CCPS Total were unexpected. Several reasons could explain this pattern. Baseline scores were already high, so on retest ratings can slip a little toward the middle. With no teaching to keep the topic in mind, interest and confidence may fade during a busy semester. On a second look, students may also answer more cautiously after reflecting on trade-offs, so strong agreement softens. End-of-term fatigue can further nudge scores down.

Although we did not measure professional identity directly, the improvements observed in Advocacy & Experience and Willingness-to-Act suggest that the module may have begun to broaden students’ perceived role beyond technical clinical delivery toward a dentist identity that includes stewardship, prevention-led care, and communication/advocacy on climate–health issues. At the same time, the generally high baseline scores and the non-significant between-group effect for the Oral Health domain indicate that many students may still locate sustainability primarily as an adjunct to clinical competence rather than a core element of professional purpose.

Our findings align with the international sustainability/planetary health education literature already cited, where brief/asynchronous interventions typically yield small–moderate gains, while longitudinal, vertically integrated approaches may support broader and more durable shifts in awareness and professional orientation. Notably, international benchmarking efforts such as the Planetary Health Report Card (Dentistry)which is a student-driven, metric-based initiative evaluating dental schools across domains including curriculum, research, community outreach/advocacy, student initiatives, and campus sustainability, illustrate how sustainability expectations are being operationalised across institutions and countries [51].

Together, these comparisons suggest that embedding assessed sustainability learning within clinically oriented programmes can contribute to an expanded graduate profile that includes prevention, stewardship, and advocacy in addition to technical competence.

This study has several limitations that should temper interpretation. Allocation by institution rather than randomization risks confounding from site culture, teaching style, staffing, or infrastructure, and although GPA differences were adjusted for, unmeasured site-level factors may persist. Outcomes were self-reported at the end of teaching, so they are vulnerable to social desirability and common-method bias, and we lacked objective behavioral or emissions metrics and any follow-up to test durability. With only two sites, cluster effects are inseparable from the intervention, and concurrent exposures outside the module could have influenced scores. We also did not assess affective responses such as climate worry or eco-anxiety; these can rise with climate education and, if unaddressed, could mediate or moderate learning and action, but their presence and direction here are unknown. Finally, the constructs at stake inckuding dental sustainability, climate change’s impacts on oral health and on general health, climate literacy, climate-resilient health systems, and mitigation versus adaptation are broad and partly distinct; a 14-week module cannot comprehensively cover or assess all relevant learning outcomes across these domains, and misalignment between some measures and the module’s emphasis may have constrained detectable change. Finally, outcomes were assessed only immediately post-teaching; we did not evaluate longer-term retention or post-graduation outcomes, so the durability of these changes and their translation into clinical practice remain to be established in future longitudinal follow-up.

Educational and policy implications follow directly. Dental schools seeking to meet emerging expectations from regulators and professional bodies can integrate sustainability and One Health competencies as assessed and credit bearing outcomes, not as extracurricular content. Constructive alignment is essential, with Objective Structured Clinical Examination (OSCE) stations, case write ups, clinical examples that require carbon aware decision making. Placement based sustainability projects using the principles of sustainability in quality improvement can anchor learning in clinic operations and generate measurable changes that matter for patients and services. Because prevention remains the dominant mitigation lever in dentistry, curricula should keep prevention at the center while teaching the systems skills that allow clinics to reduce travel, energy and water demand, and waste without compromising safety and equity.Parallel continuing professional development for clinical tutors and staff can reinforce role modelling and reduce hidden curriculum effects that may otherwise dampen advocacy and perceived feasibility.

## Conclusion

A semester-long, embedded module in environmental sustainability improved multiple dimensions of climate–health literacy and action readiness among dental students, with the largest gains among those with lower baseline scores, as indicated by the baseline-by-group interaction and simple-effects estimates (Table 5). This gap-narrowing pattern is consistent with scaffolded and mastery-oriented learning, which provides greatest benefit to learners furthest from the intended competence, and with reduced headroom/ceiling effects among students starting at higher levels. Oral Health specific perceptions were high at baseline and showed only modest growth. Taken together with the wider evidence base on climate and health education in dentistry and other health professions, these findings support a shift from brief add-ons toward integrated, assessed, and practice connected and widely adopted curriculums that prepare future dentists to deliver high value, climate friendly oral health care and to lead change within clinics and communities.

## Supplementary Information

Supplementary Material 1.

Supplementary Material 2.

## Data Availability

De-identified datasets can be accessed online on Mendeley open data repository through DOI:10.17632/nkv4pnsmy5.1.
