# Supplementary material for: Strengthening climate-health literacy through sustainability education among dental students: a quasi-experimental evaluation
Source: BMC Med Educ. 2026 Mar 11;26:633. doi: 10.1186/s12909-026-08987-1 (PMC13088611; doi:10.1186/s12909-026-08987-1)
Supplement: Supplementary file 1 — Supplementary Material 1. [file 12909_2026_8987_MOESM1_ESM.docx]

**Ağız ve Diş Sağlığı**

31. İklim değişikliği,  su kaynaklarının miktar, hijyen ve kalitesini etkileyerek ağız ve diş hijyenini sürdürmekte problem yaratabilir. (Climate Change can create problems for maintaining oral hygiene bu affecting the quantity, quality and hygiene of water sources.)

32. İklim değişikliği solunum yolu hastalıklarının riskini arttırarak ağız kuruluğuna sebep olabilir, bu da ağız ve diş sağlığı için tehdittir. (Climate Change can increase the risk of respiratory diseases causing dry mouth, that is a threat for oral health.)

33. İklim değişikliği ve gıda güvenliği arasındaki ilişki nedeniyle malnutrisyon sonucu toplumda görülen mine hipoplazilerinin artma riski vardır. (Due to the relationship between climate change and food security, there is a risk of an increase in enamel hypoplasia in the population as a result of malnutrition)

34. İklim değişikliği ağız kanserlerini arttırabilir. (Climate Change can increase oral cancers.)

35. İklim değişikliği içme sularıdaki florür miktarını takip etmeyi zorlaştırarak florozis ya da çürük insidansını arttırabilir. (Climate change may increase the incidence of fluorosis or caries by making it more difficult to monitor the amount of fluoride in drinking water.)

36. Diş tedavilerinin çevreye verdiği zararı azaltmak için geri dönüşüm en etkili yöntemdir. (Recycling is the most effective method to reduce the environmental damage caused by dental treatments.)

**Bilinçten Eyleme**

37. İklim değişikliğinin sağlık üzerindeki etkileri hakkında yeterli bilgiye sahibim. (I have sufficient knowledge about the effects of climate change on health.)

38. İklim değişikliğinin sağlık etkilerini azaltmada kişisel sorumluluğum olduğuna inanıyorum. (I believe I have a personal responsibility to reduce the health impacts of climate change.)

39. İklim değişikliği ile ilgili daha sürdürülebilir sağlık uygulamaları için adımlar atmayı planlıyorum. (I plan to take steps for more sustainable health practices related to climate change.)

40. Diş hekimliği uygulamalarımda sürdürülebilirlik açısından değişiklikler yapmaya hazırım (I am ready to make changes in my dental practice in terms of sustainability.)

41. Sağlık çalışanı olarak toplumu iklim değişikliğinin sağlık üzerindeki etkileri konusunda bilgilendirme konusunda aktif rol almak isterim. (As a health worker, I would like to take an active role in informing the public about the effects of climate change on health.)

42. Sürdürülebilir sağlık uygulamaları ile ilgili eğitimler ve bilgilendirme toplantıları, benim bu alanda daha fazla adım atmamı sağlar. (Trainings and information meetings on sustainable health practices enable me to take further steps in this field.)

43. İklim değişikliğinin sağlık etkilerini azaltmaya yönelik eylemlerde bulunmam, günlük iş yoğunluğum ve pratik zorluklar nedeniyle sınırlıdır. (My ability to take action to reduce the health impacts of climate change is limited by my daily workload and practical difficulties.)
