# Supplementary material for: Strengthening climate-health literacy through sustainability education among dental students: a quasi-experimental evaluation
Source: BMC Med Educ. 2026 Mar 11;26:633. doi: 10.1186/s12909-026-08987-1 (PMC13088611; doi:10.1186/s12909-026-08987-1)
Supplement: Supplementary file 2 — Supplementary Material 2. [file 12909_2026_8987_MOESM2_ESM.docx]

**1. Course Identification**

| **Field** | **Information** |
| --- | --- |
| Course Title (English) | **Biomimetic Restorative Approaches for Sustainable Dental Practice** |
| Course Title (Turkish, official) | Sürdürülebilir Diş Hekimliği Pratiği için Biomimetik Restoratif Yaklaşımlar |
| Course Code | (to be assigned) |
| Programme | Dentistry (Undergraduate) |
| Level of Course | First Cycle (Bachelor / DDS) (One tier programme) |
| Year / Semester | 4th year, Spring semester |
| Type of Course | Elective |
| Language of Instruction | English |
| Mode of Delivery | Face-to-face |
| Duration | 14 weeks |
| Contact Hours | 1 hour / week |
| Local Credit | 1 |
| ECTS Credits | 2 |
| Course Coordinator | Prof. Sema Belli, Department of Endodontics, Faculty of Dentistry |

**2. Course Description**

This one-semester, 14-week course “Biomimetic Restorative Approaches for Sustainable Dental Practice”integrates planetary health, climate and environmental sustainability with biomimetic restorative dentistry.

**3. Course Aims**

The course aims to enable students to:

1. Understand major planetary health and environmental challenges, including climate change, wildfires, air and water pollution and antimicrobial resistance, and their implications for general and oral health and dental services.
2. Recognise how dental care systems contribute to environmental pressures and how sustainable dentistry can reduce the environmental footprint while maintaining quality and patient safety.
3. Understand the principles and philosophy of biomimetic restorative dentistry and how reproducing natural tooth structure and function supports long-term clinical outcomes and sustainability.
4. Select and apply biomimetic restorative materials and techniques in a way that conserves tooth structure, improves restoration longevity and uses resources more efficiently.
5. Integrate planetary health, One Health, sustainability and biomimetic concepts into everyday clinical decision-making, treatment planning and communication with patients and the dental team.
6. Develop and present realistic improvement ideas or case-based examples that combine sustainability and biomimetic restorative approaches in different dental disciplines.

**4. Teaching and Learning Methods**

- Interactive lectures
- Small-group discussions and case/scenario-based learning
- Guided reading and short quizzes or polls
- Group tasks on sustainable and biomimetic treatment planning
- Case-based discussions and short student presentations

**5. Weekly Course Content (14 Weeks)**

| **Week** | **Topic / Content Title** | **Brief Description** |
| --- | --- | --- |
| 1 | Introduction to Sustainability and Sustainable Dentistry | Definitions of sustainability and sustainable development; introduction to planetary health and One Health; overview of sustainable approaches in dentistry; international and national policy context; explanation of course organisation |
| 2 | Climate Change, Climate Justice and Health | Basic climate science; climate change as a global health and oral-health challenge; examples of climate-related risks; concepts of climate justice and vulnerable populations; discussion of how these issues may affect dental patients, access to services and dental facilities. |
| 3 | Disasters, Wildfires, Extreme Weather and Air Quality | Types of climate-related disasters (wildfires, floods, storms, heat waves); effects on air quality, respiratory health and service continuity; implications for dental clinics including preparedness, scheduling, communication with patients and occupational health and safety. |
| 4 | Air and Water Pollution, Pesticides, Antimicrobial Resistance (AMR) and One Health | Sources and health effects of air and water pollution; pesticide and chemical exposure; AMR as a One Health problem; links between environmental exposures, oral microbiota and oral diseases; implications for antibiotic stewardship, infection control and dental material selection. |
| 5 | Environmental “Hot Spots” in Dental Care | Identification of major contributors to the environmental footprint of dental care: patient and staff travel, energy and water use, materials and instruments, packaging and waste, anaesthetic gases and pharmaceuticals; introduction to life-cycle thinking in dental procedures; strategies to reduce environmental footprint. |
| 6 | Sustainable Clinical Practice Across Dental Disciplines | Examples of sustainable clinical and organisational approaches in restorative dentistry, endodontics, prosthodontics, paediatric dentistry, orthodontics, periodontology, oral and maxillofacial radiology and oral and maxillofacial surgery; appointment planning; waste segregation and recycling; maintaining quality and safety. |
| 7 | Quality Improvement, 4R Approach, Communication and Advocacy | Basic quality-improvement concepts in dental practice; the 4R approach (Reduce, Reuse, Recycle, Rethink) and its application in dental settings; identifying opportunities for improvement in dental school/clinic; communication with patients and colleagues; advocacy roles of dentists; review before the midterm exam. |
| 8 | Introduction to Biomimetic Restorative Dentistry | Concept and philosophy of biomimetic dentistry; structure and function of enamel, dentine and the dentino-enamel junction; viewing the tooth as a biomechanical organ; how biomimetic approaches aim to reproduce natural tooth properties; links between biomimetic principles, tooth survival and environmental sustainability. |
| 9 | Tooth Biomechanics and Adhesion in a Biomimetic Perspective | Biomechanics of intact and restored teeth; stress distribution, crack formation and propagation, fatigue and fracture; fundamentals of adhesion and the hybrid layer; relationship between adhesive performance, restoration longevity and reduced need for replacement; clinical and environmental implications. |
| 10 | Biomimetic Restorative Materials and Their Sustainable Use | Overview of biomimetic restorative materials (adhesive systems, resin composites, glass ionomer-based materials, fibre-reinforced materials and other adhesive options); indications, advantages and limitations; considerations regarding durability, biocompatibility and possible environmental impacts (production, packaging, waste). |
| 11 | Minimally Invasive Biomimetic Techniques I: Vital Teeth | Clinical decision-making for minimally invasive, biomimetic restorations in vital teeth; management of occlusal and proximal lesions; deep caries management and selective caries removal; preservation of pulp vitality; layering strategies and control of polymerisation stress; sustainability benefits of reduced invasiveness. |
| 12 | Minimally Invasive Biomimetic Techniques II: Endodontically Treated Teeth | Biomimetic restoration of endodontically treated teeth; indications for partial cuspal coverage, onlays and overlays; use of fibre posts and adhesive cores; criteria for avoiding full-coverage crowns when possible; evidence on tooth survival and fracture resistance; impact on retreatment rates, material use and sustainability. |
| 13 | Case-Based Decision-Making: Biomimetic vs Conventional Approaches | Discussion of clinical cases comparing biomimetic and conventional restorative options; integration of patient factors, tooth prognosis, cost, aesthetics, longevity and environmental considerations; design of biomimetic–sustainable treatment plans in small groups; reflection on decision-making in daily practice. |
| 14 | Student Presentations and Course Integration | Short student presentations of selected cases or clinic-based ideas combining planetary health concepts, sustainable dentistry and biomimetic restorative approaches; group feedback and discussion; integration of learning from all 14 weeks; reflection on professional identity and willingness to act; preparation for the final exam. |

**6. Assessment and Grading**

| **Assessment Type** | **Component** | **Number** | **Weight (%)** |
| --- | --- | --- | --- |
| In-term Assessment | Midterm exam (written) | 1 | 50 |
| End-of-term Assessment | Final exam (written) | 1 | 50 |
|  | **Total** |  | **100** |

Short quizzes, participation and group tasks may be used as formative assessments without separate numerical weight if not required by faculty regulations.

**7. Workload and ECTS**

| **Activity** | **Number of Weeks / Activities** | **Duration (hours)** | **Total Workload (hours)** |
| --- | --- | --- | --- |
| Weekly lecture | 14 | 1 | 14 |
| Weekly reading and preparation | 14 | 2 | 28 |
| Preparation for midterm exam | 1 | 3 | 3 |
| Preparation for final exam | 1 | 5 | 5 |
| **Total Workload** |  |  | **50** |

**ECTS Credits (suggested):** 50 total hours ÷ 25 = **2 ECTS**

**8. Course Learning Outcomes (CLOs)**

| **Code** | **Course Learning Outcome** |
| --- | --- |
| CLO1 | Explain the basic mechanisms of climate change and describe how climate change, environmental degradation and climate-related disasters (such as wildfires, floods and heat waves) affect general health and oral health. |
| CLO2 | Describe planetary health and One Health concepts and explain the two-way relationships between dental practice, environmental systems and population health. |
| CLO3 | Identify the main environmental “hot spots” of dental services (e.g. patient and staff travel, energy use, materials and consumables, chemicals and waste) and discuss their contribution to greenhouse gas emissions and environmental pollution. |
| CLO4 | Discuss how air and water pollution, pesticide exposure, antimicrobial resistance and other environmental risk factors influence oral diseases and oral-health-care delivery, particularly for vulnerable populations. |
| CLO5 | Describe the principles of biomimetic restorative dentistry and explain how preserving tooth structure, reproducing natural biomechanics and improving restoration longevity contribute to both clinical quality and environmental sustainability. |
| CLO6 | Select and justify appropriate biomimetic restorative materials and techniques for common clinical scenarios, balancing biological, mechanical, aesthetic and environmental considerations. |
| CLO7 | Use the 4R approach (Reduce, Reuse, Recycle, Rethink) and basic quality-improvement thinking to outline a low-carbon, resource-efficient, biomimetic pathway in at least one dental discipline (e.g. restorative, endodontic or prosthodontic practice). |
| CLO8 | Communicate clearly with patients and the dental team about sustainable and biomimetic treatment options and present a small case-based or clinic-based idea that demonstrates willingness to act for sustainable dental practice. |

**9. Programme Outcomes (POs) – Dentistry**

| **Code** | **Programme Outcome** |
| --- | --- |
| PO1 | Understands growth and development, recognises orthodontic anomalies, carries out basic preventive and interceptive orthodontic interventions where appropriate and refers patients for specialist treatment at the correct time. |
| PO2 | Has sufficient knowledge in basic and clinical sciences to understand normal and pathological oral and dental conditions; gives special importance to infection control and can ensure safe working conditions; is trained to identify the patient’s main complaint. |
| PO3 | Plans and applies space maintainers and simple appliances to move a single tooth or correct cross-bite, where appropriate. |
| PO4 | Develops and implements effective strategies to prevent or manage dental and medical emergencies that may be encountered in practice. |
| PO5 | Communicates effectively with other members of the dental team; understands ethical principles in dentistry; demonstrates personal and professional integrity and respects patients and colleagues regardless of language, culture, gender, race or social status. |
| PO6 | Knows dental biomaterials and restorative materials, their indications, limitations and possible environmental impacts; recognises when medical consultation or referral to another specialist is required and initiates this process appropriately. |

**10. CLO–PO Alignment Matrix**

**Scale:** 0 = No contribution, 1 = Low, 2 = Moderate, 3 = High

| **CLO \ PO** | **PO1** | **PO2** | **PO3** | **PO4** | **PO5** | **PO6** |
| --- | --- | --- | --- | --- | --- | --- |
| CLO1 – Climate change & health–oral health links | 1 | 3 | 0 | 2 | 2 | 1 |
| CLO2 – Planetary / One Health concepts | 1 | 3 | 0 | 1 | 2 | 2 |
| CLO3 – Environmental hot spots in dental care | 1 | 2 | 0 | 1 | 2 | 3 |
| CLO4 – Environmental risks (air/water pollution, pesticides, AMR) & oral health | 1 | 3 | 0 | 2 | 2 | 2 |
| CLO5 – Biomimetic principles & sustainability | 1 | 3 | 0 | 1 | 2 | 3 |
| CLO6 – Material and technique selection in biomimetic restorative care | 1 | 3 | 0 | 2 | 2 | 3 |
| CLO7 – 4R approach & biomimetic low-carbon workflows | 1 | 2 | 1 | 2 | 2 | 3 |
| CLO8 – Communication, advocacy & case-based sustainability / biomimetic idea | 1 | 2 | 0 | 2 | 3 | 2 |
